# Highly Sensitive Textile-Based Capacitive Pressure Sensors Using PVDF-HFP/Ionic Liquid Composite Films

**DOI:** 10.3390/s21020442

**Published:** 2021-01-09

**Authors:** Kyobin Keum, Jae Sang Heo, Jimi Eom, Keon Woo Lee, Sung Kyu Park, Yong-Hoon Kim

**Affiliations:** 1 School of Advanced Materials Science and Engineering, Sungkyunkwan University, Suwon 16419, Korea; fiete1202@skku.edu (K.K.); heojs38@gmail.com (J.S.H.); 2 Advanced Textile R&D Department, Research Institute of Convergence Technology, Korea Institute of Industrial Technology (KITECH), Ansan 15588, Korea; jimi0321@naver.com; 3 School of Electrical and Electronic Engineering, Chung-Ang University, Seoul 06974, Korea; lkw9941@gmail.com; 4 SKKU Advanced Institute of Nanotechnology (SAINT), Sungkyunkwan University, Suwon 16419, Korea

**Keywords:** electronic textiles, capacitive pressure sensors, PVDF-HFP, ionic liquid, contact area

## Abstract

Textile-based pressure sensors have garnered considerable interest in electronic textiles due to their diverse applications, including human–machine interface and healthcare monitoring systems. We studied a textile-based capacitive pressure sensor array using a poly(vinylidene fluoride)-co-hexafluoropropylene (PVDF-HFP)/ionic liquid (IL) composite film. By constructing a capacitor structure with Ag-plated conductive fiber electrodes that are embedded in fabrics, a capacitive pressure sensor showing high sensitivity, good operation stability, and a wide sensing range could be created. By optimizing the PVDF-HFP:IL ratio (6.5:3.5), the fabricated textile pressure sensors showed sensitivity of 9.51 kPa^−1^ and 0.69 kPa^−1^ in the pressure ranges of 0–20 kPa and 20–100 kPa, respectively. The pressure-dependent capacitance variation in our device was explained based on the change in the contact-area formed between the multi-filament fiber electrodes and the PVDF-HFP/IL film. To demonstrate the applicability and scalability of the sensor device, a 3 × 3 pressure sensor array was fabricated. Due to its matrix-type array structure and capacitive sensing mechanism, multi-point detection was possible, and the different positions and the weights of the objects could be identified.

## 1. Introduction

Electronic textiles (e-textiles) have recently garnered considerable interest in the field of wearable electronics due to the diverse applications that are enabled by integrating them into various sensor devices [1,2,3], triboelectric nanogenerators [4,5,6], wireless communication devices [7,8,9], logic circuits [10], and control systems [11]. Unlike conventional electronic devices, e-textiles can be made virtually unnoticeable by fully embedding them in clothes or fabrics. Therefore, applications requiring an ‘always-on’ mode operation can be more effectively performed by using e-textiles. Among various electronic components that can be integrated into e-textiles are strain sensors [12,13], motion sensors [14], environmental biosensors [15,16], and pressure sensors [17,18]. In particular, the integration of pressure sensors has been widely investigated to broaden the applications of e-textiles in breath monitoring [19], heart monitoring [20], and human-machine interface systems [21,22,23]. In these applications, the required pressure sensitivity and pressure-detection ranges are varied. For example, in breath and heart monitoring, pressure sensors should have a high sensitivity in the low-pressure range [24]; while in applications such as body-weight monitoring, sensors should have higher detection range and better sensitivity in the high-pressure range [25]. From this perspective, pressure sensors that possess both high sensitivity and a wide sensing range are in high demand in e-textile applications.

There are several types of mechanisms for pressure detection. These include resistive, piezoresistive, piezoelectric, and capacitive types [26]. The capacitive-type pressure-sensing mechanism can especially provide advantages such as multi-point detection and low power consumption [11,27,28,29]. Considering that e-textiles typically require low-power operation due to the limitation of their power supply, capacitive-type pressure sensors can be a good candidate for e-textiles. For the realization of textile-based capacitive pressure sensors, various device structures and materials have been investigated. For example, Li et al. demonstrated a capacitive pressure sensor based on a honeycomb-weaving architecture that exhibited a sensitivity of 0.045 kPa^−1^ under 10 kPa [30]. Vu et al. studied a capacitive pressure sensor using polyester/spandex (PET/SP) fabrics with a combination of single-walled carbon nanotubes and stretchable silver ink [25]. The fabricated sensor exhibited a sensitivity of 0.02–0.042 kPa^−1^ depending on the number of encapsulation lines. In another approach, Choi et al. studied a porous elastomer/multiwalled carbon nanotube composite as a sensing material that exhibited a pressure sensitivity of 6.42 kPa^−1^ and 1.72 kPa^−1^ in the range of 0–2 kPa and 2–10 kPa, respectively [31]. Wu et al. utilized silver nanofiber-coated conductive fabrics and a spacer fabric to construct a textile pressure sensor [32] that exhibited a sensitivity of 0.283 kPa^−1^ and good operational stability. Although these previous investigations are noteworthy, the pressure sensitivities were relatively low, and the detection ranges were somewhat limited. Among various material candidates for capacitive pressure sensors, ionic liquid (IL)-based soft ion-gel films are of significant interest due to their high dielectric constants. Ion-gel films have been frequently adopted as a gate dielectric layer in thin-film transistors to reduce the operation voltage and enhance the electrical properties [33,34,35]. In capacitive pressure sensors, the high-k ion-gel film can also enhance their response to the applied pressure and their sensitivity [36]. In addition, ion-gel films possess good mechanical flexibility and stability, which could enable their integration in e-textile devices.

In this study, we demonstrate a textile-based capacitive pressure sensor using a poly(vinylidene fluoride)-co-hexafluoropropylene (PVDF-HFP)/IL composite film. The PVDF-HFP/IL film is used as a dielectric layer in the capacitor, positioned between two fabric sheets with multi-filament Ag-plated fiber electrodes. The Ag-plated fibers and the PVDF-HFP/IL film formed a cross-point capacitor structure in which its capacitance value varies with the applied pressure. The fabricated capacitive pressure sensor exhibited high sensitivity, good stability, and wide sensing range. Particularly, with optimized PVDF-HFP:IL ratio (6.5:3.5), the sensor showed a sensitivity of 9.51 kPa^−1^ and 0.69 kPa^−1^ in the pressure ranges of 0–20 kPa and 20–100 kPa, respectively. The response times of the sensor under loading and unloading conditions were −0.8 s and −0.5 s, respectively. The pressure-dependent capacitance variation is explained based on the change in the contact area, which is formed between the multi-filament fiber electrodes and the PVDF-HFP/IL film. To demonstrate the applicability and scalability of the sensor device, we used a 3 × 3 pressure sensor array. Due to its matrix-type array structure and the capacitive sensing mechanism, multi-point detection was possible, and it successfully identified the different positions and weights of the objects.

## 2. Materials and Methods

**Figure 1:**
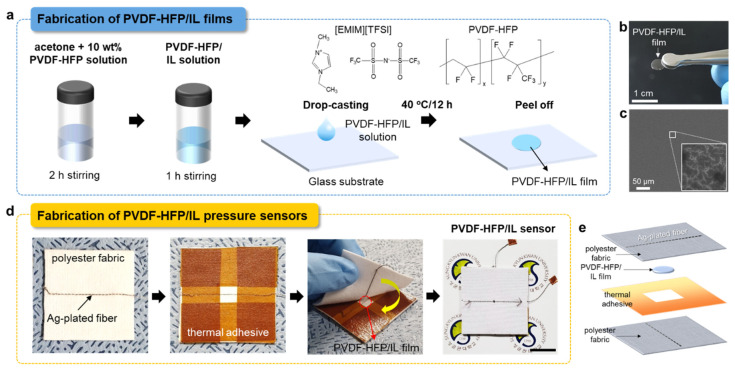
(**a**) The fabrication process of the PVDF-HFP/IL film using the drop-casting method and the molecular structures of [EMIM][TFSI] and PVDF-HFP. (**b**) An optical image, and (**c**) an FESEM image of PVDF-HFP/IL film (PVDF-HFP:IL = 6.5:3.5). (**d**) The fabrication process of PVDF-HFP/IL-based textile pressure sensor (scale bar: 2 cm). (**e**) A schematic structure of the PVDF-HFP/IL-based textile pressure sensor.

The fabrication process of PVDF-HFP/IL film is shown in Figure 1a. To fabricate the PVDF-HFP/IL film, a drop-casting method was used. To prepare the PVDF-HFP solution, PVDF-HFP pellets (average M_w_ −455,000, Sigma Aldrich, St. Louis, MO, USA) were dissolved in acetone at 10 wt % and thoroughly stirred for 2 h. Then, the PVDF-HFP solution and 1-ethyl-3-methylimidazolium bis(trifluoromethylsulfonyl)imide ([EMIM][TFSI]) IL (≥98%, M_w_ = 391.31, purchased from Sigma Aldrich) were mixed at different weight ratios and stirred for −1 h. The weight ratios of PVDF-HFP:IL varied as 5:5, 6:4, and 6.5:3.5. To fabricate a freestanding PVDF-HFP/IL film, which is used as a dielectric layer in the textile pressure sensor, a 3 cm × 3 cm bare glass substrate was prepared and thoroughly cleaned with acetone, isopropanol alcohol, and deionized water, then dried with dry nitrogen. The PVDF-HFP/IL solution was dropped on the cleaned glass substrate using a micropipette with a dropping volume of −10 μL. Next, to cure the film, a thermal annealing at 40 °C was carried out for 12 h. After the curing, the film was detached from the glass substrate. The average diameter of the fabricated PVDF-HFP/IL film was −0.5 cm. To fabricate the pressure sensor, Ag-plated conductive nylon fibers (fiber diameter: 280d, SOITEX, Goyang, Korea) was stitched on both the top and bottom polyester fabric sheets, which had dimensions of 5 cm × 5 cm. A double-sided thermoplastic adhesive film (Thermal Bonding Film 583, 3M, Maplewood, MN, USA) was then bonded to the bottom polyester fabric sheet by applying 2.56 kPa of pressure at 110 °C for 10 min. To expose the Ag-plated fiber electrode, which was located at the center region of the fabric sheet, the center region of the adhesive film was cut to a dimension of −0.5 cm × 0.5 cm. Then, the free-standing PVDF-HFP/IL film was placed on top of the Ag-plated fiber electrode. Next, a top fabric sheet having Ag-plated fiber electrode was covered, and finally the sample was hot pressed for 1 min by applying 2.56 kPa of pressure at 60 °C to bond the fabric sheets together.

The surface morphology of the PVDF-HFP/IL film was analyzed using a field-emission scanning electron microscope (FESEM) (JSM-7600F, JEOL, Tokyo, Japan). To measure the pressure-sensing characteristics, a force gauge (M5-2, MARK-10, Copiague, NY, USA) was utilized. The measured pressure range was 0–100 kPa. The characterizations of the pressure sensor were carried out using a probe station (model 8000, MS-TECH, Hwaseong, Korea) connected to an LCR meter (4284A, Agilent Technologies, Inc., Santa Clara, CA, USA) and a semiconductor parameter analyzer (4200A-SCS, Keithley, Cleveland, OH, USA). The capacitance value of the pressure sensor was measured with an applied voltage of 1 V at frequencies of 0.1–100 kHz.

## 3. Results

### 3.1. The Device Structure and Electrical Characterization of PVDF-HFP/IL Films

The optical and FESEM images of the PVDF-HFP/IL film are shown in Figure 1b,c, respectively (PVDF-HFP:IL ratio of 6.5:3.5). As shown in Figure 1c, the PVDF-HFP:IL film had a granular-like morphology, showing small micro-cracks on the film surface. Using the PVDF-HFP:IL film, a textile-based capacitive pressure sensor was fabricated following the procedure described in Figure 1d. Figure 1e shows the schematic structure of the pressure sensor. To form a capacitor structure, the PVDF-HFP/IL film was sandwiched between top and bottom fabric sheets with multi-filament Ag-plated conducting fibers. The electrical resistance of the Ag-plated conducting fiber was about 1 Ω/cm, and the top and bottom Ag-plated fibers were positioned orthogonally to form a cross-point structure. To firmly attach the two fabric sheets, a thermal adhesive film was inserted between the fabric sheets and hot-pressed. As described, the PVDF-HFP/IL film played important roles in our sensor device, as a dielectric layer for capacitor formation and as a basis for capacitance variation under applied pressure. The PVDF-HFP/IL film was composed of PVDF-HFP and IL; the PVDF-HFP formed a network structure, and the ILs contributed to the formation of the electric double layer (EDL). Among various available polymer materials, PVDF-HFP was selected as the matrix polymer due to its good mechanical stability and easy film-forming ability due to fluorination and its low surface energy [37,38]. PVDF-HFP has good thermal stability, good electrochemical stability, low crystallinity, and high dielectric constant (ε = 8.4) [39]; therefore, the composite of PVDF-HFP and IL with high ionic conductivity could be effective as a sensing material in capacitive-type pressure sensors. Under an applied bias, the IL molecules migrated and redistributed near the electrodes and formed EDLs. Since the amount of ILs in the PVDF-HFP/IL film had a strong influence on the initial capacitance value and the pressure sensitivity of the sensor, it was necessary to optimize the ratio of PVDF-HFP and IL in the film. In our study, the PVDF-HFP:IL ratio was varied as 6.5:3.5, 6:4, and 5:5. When the IL ratio was too high (typically higher than 5), a gel-like film was obtained instead of a dense free-standing film, due to the lack of PVDF-HFP component in the film. As a consequence, detaching the film from the glass substrate and handling the film were difficult. However, when the IL ratio was lower than 3.5, the viscosity of the solution was considerably increased, and when the film was cured, an abnormal cone shape structure was formed at the center region of the film, possibly due to the high viscosity of the PVDF-HFP/IL solution. This could prevent the formation of uniform contacts between the Ag-plated fiber electrodes and the PVDF-HFP/IL film. Therefore, in our experiment, the PVDF-HFP:IL ratio was set in the range of 6.5:3.5–5:5.

**Figure 2:**
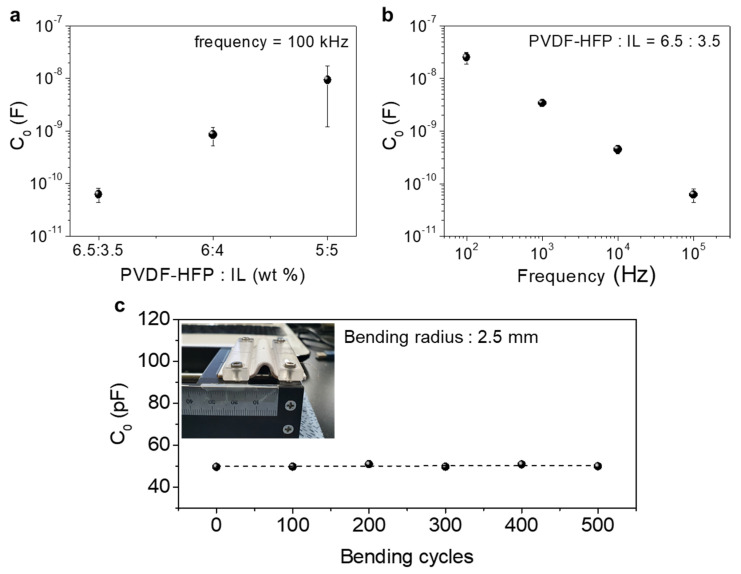
(**a**) The variation of initial capacitance as a function of the PVDF-HFP:IL ratio (frequency = 100 kHz). (**b**) The variation of initial capacitance as a function of the frequency (PVDF-HFP:IL = 6.5:3.5). (**c**) The initial capacitance (C_0_) variation of the PVDF-HFP/IL textile pressure sensor during the cyclic bending test (500 bending cycles, bending radius of 2.5 mm). The inset shows an optical image of the textile pressure sensor under bent condition.

To investigate the effects of PVDF-HFP:IL ratio on the initial capacitance (C_0_) value of the film, PVDF-HFP/IL films with different ratios were prepared. Figure 2a shows the change of C_0_ as a function of PVDF-HFP:IL ratio. When the PVDF-HFP:IL ratio was varied as 6.5:3.5, 6:4 and 5:5, the C_0_ increased to 0.062 nF, 0.85 nF and 9.32 nF, respectively. The increased C_0_ can be attributed to the larger amount of ILs in the PVDF-HFP/IL film, which contributed to the EDL formation at the interfaces with the top and bottom fiber electrodes. According to the Stern theory [40], the ions of opposite polarity to the electrode move toward the electrode, forming a Stern layer near the interface. In addition, the ions that had the same polarity as the electrode moved away from the interface and diffused into the film to form a diffusion layer [40]. Due to the formation of EDLs at both electrodes that had an opposite polarity, a high capacitance was induced. One of the unique characteristics of an IL-based dielectric layer is a large frequency-dependent capacitance variation [36,41] due to the relatively slow migration of ILs compared to that of dipole polarization occurring in typical inorganic dielectric films [36,41]. Figure 2b shows the change of initial capacitance value as a function of the frequency. As displayed, the C_0_ value decreased from −25 nF to 0.062 nF when the frequency was increased from 100 Hz to 100 kHz. In our study, because the initial capacitance value directly affected the pressure sensitivity (S) as shown in the following equation:

(1) $$S=\frac{∆C/C_{0}}{∆P}=\frac{\left(C-C_{0}\right)/C_{0}}{P-P_{0}}$$

where P is the applied pressure, optimization of the operation frequency was necessary. Additionally, the mechanical stability of PVDF-HFP/IL film is important in achieving highly stable devices. To evaluate the mechanical stability of PVDF-HFP/IL film, we performed a cyclic bending test of the pressure sensor (500 cycles at a bending radius of 2.5 mm) and traced the relative change of the C_0_ value. As shown in Figure 2c, the C_0_ value was only changed by 2.7% after the bending test, indicating that the PVDF-HFP/IL film had good mechanical stability.

### 3.2. Pressure-Sensing Characteristics of PVDF-HFP/IL-Based Textile Pressure Sensors

**Figure 3:**
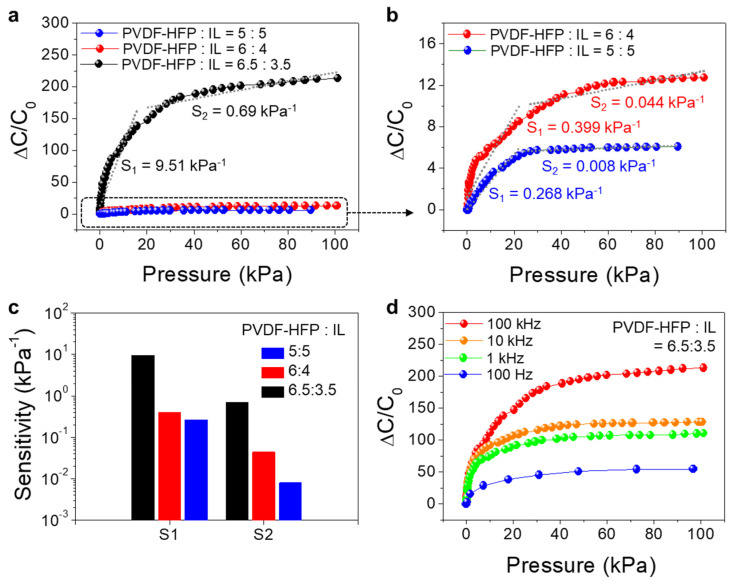
(**a**,**b**) Pressure-sensing characteristics (ΔC/C_0_ vs. P) of PVDF-HFP/IL-based pressure sensors fabricated with different PVDF-HFP:IL ratios (5:5 (blue), 6:4 (red), and 6.5:3.5 (black). (**c**) The pressure sensitivities in the low-pressure (0–20 kPa) (S_1_) and high-pressure (20–100 kPa) regions. S_1_ and S_2_ represent the sensitivity values at low pressure and high pressure, respectively. (**d**) Frequency-dependent pressure-sensing characteristics of the PVDF-HFP/IL-based pressure sensors (PVDF-HFP:IL ratio = 6.5:3.5).

To examine the effects of the PVDF-HFP:IL ratio on sensing characteristics such as pressure sensitivity and detection range, textile-based pressure sensors using PVDF-HFP/IL films with different PVDF-HFP:IL ratios were fabricated. Figure 3a shows the plots of ΔC/C_0_ vs. P measured from each pressure sensor at a frequency of 100 kHz. The capacitance variation (ΔC/C_0_) was largely dependent on the PVDF-HFP:IL ratio, and particularly, the sensors fabricated with PVDF-HFP:IL ratio of 6.5:3.5 exhibited the highest capacitance change, while those fabricated with PVDF-HFP:IL ratios of 5:5 and 6:4 showed much reduced capacitance variations (Figure 3b). Figure 3c summarizes the pressure sensitivities obtained in the ranges of 0–20 kPa (S_1_) and 20–100 kPa (S_2_). When the PVDF-HFP:IL ratio was 6.5:3.5, the S_1_ and S_2_ were 9.51 kPa^−1^ and 0.69 kPa^−1^, respectively. However, as the IL weight ratio was increased, the S_1_ and S_2_ decreased substantially. One of the main reasons for the reduced sensitivity was the high C_0_ values of the film (Figure 2a). Specifically, the C_0_ value increased by one to two orders of magnitude when the PVDF-HFP:IL ratio was changed from 6.5:3.5 to 6:4 and 5:5. In all samples, there was a considerable transition of pressure sensitivity at around P—20 kPa. It was expected that at this pressure value, the dominant mechanism for the capacitance variation would change. In the low-pressure range, the variation of the contact area that formed between the multi-filament fiber electrodes and the PVDF-HFP/IL film was dominant, and induced a large capacitance change. In contrast, in the high-pressure range, the contact-area variation became less dominant, and the structural deformation of the PVDF-HFP/IL film became more dominant. Based on these results, we claim that the sensor fabricated with PVDF-HFP:IL ratio of 6.5:3.5 had a wider detection range and a higher sensitivity.

Additionally, because the capacitance of PVDF-HFP/IL film is also varied by the frequency, the frequency-dependent pressure-sensing characteristics were evaluated. As shown in Figure 3d, as the frequency decreased from 100 kHz to 0.1 kHz, the capacitance variation (ΔC/C_0_) by the applied pressure decreased, which can be also attributed to the higher C_0_ values at lower frequencies (Figure 2b). More importantly, the response at high pressure (S_2_) was relatively low when the frequency was 0.1–10 kHz, limiting the detection range of the sensor. In overall, the results suggest that a PVDF-HFP:IL ratio of 6.5:3.5 and an operation frequency of 100 kHz are the most suitable to achieve high-pressure sensitivity and a wider pressure-detection range.

### 3.3. Pressure-Sensing Mechanism

**Figure 4:**
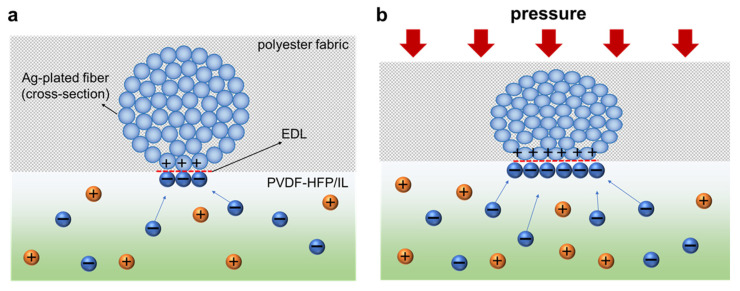
Pressure-sensing mechanism of the PVDF-HFP/IL-based pressure sensor. (**a**) Without an external pressure, the contact area that formed between the fiber electrode and the PVDF-HFP/IL film was relatively small, resulting in lower EDL formation. (**b**) With an external pressure, the contact area and the EDL formation region were extended, increasing the capacitance value.

In the PVDF-HFP/IL textile pressure sensor, the two multi-filament Ag-plated fiber electrodes and the PVDF-HFP/IL dielectric film formed a capacitor. Therefore, the capacitance can be expressed by the following equation:

(2) $$C=\varepsilon \frac{A}{d}$$

where *ε* is permittivity of the dielectric, *A* is the area, and *d* is the thickness of the dielectric film. Then, by applying an external pressure, the area and the thickness of the dielectric film can be changed, thereby increasing the capacitance. In our device structure, it was suggested that although the thickness of PVDF-HFP/IL film was decreased by pressure, the increase of contact area made between the multi-filament fiber electrodes and the PVDF-HFP/IL film was considered as the dominant mechanism for the capacitance variation [36], particularly at low pressures. When there was no external force, the contact area made between the fiber electrodes and the PVDF-HFP/IL film was small where the EDL can be formed (Figure 4a). However, as the applied pressure was increased, the multi-filament fiber structure was deformed and pushed into the PVDF-HFP/IL film, which increased the contact area of the EDL formation (Figure 4b). As a result, the capacitance value increased with the applied pressure. However, after a certain level of pressure, in our case at around P—20 kPa, the rate of contact-area increase was reduced, and the capacitance variation was also reduced, resulting in lower sensitivity (Figure 3a,b).

### 3.4. Response Time and the Stability of PVDF-HFP/IL Pressure Sensors

**Figure 5:**
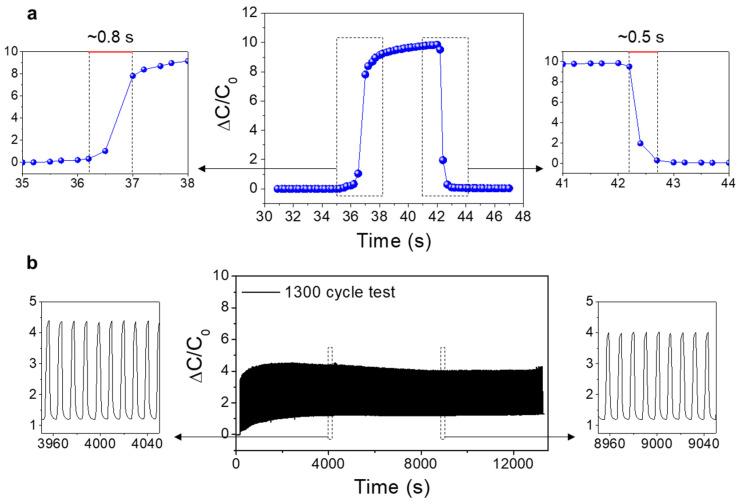
(**a**) The response characteristics of the PVDF-HFP/IL textile pressure sensor (PVDF-HFP:IL ratio = 6.5:3.5, loading pressure = 2 kPa). The response times for loading and unloading conditions were −0.8 s and −0.5 s, respectively. (**b**) The cyclic test results for the PVDF-HFP/IL textile pressure sensor (1300 cycles, loading pressure = 1 kPa).

The response characteristics of the pressure sensor are also important parameters for determining the applications of the sensor. Figure 5a shows the response characteristics of PVDF-HFP/IL textile pressure sensor (PVDF-HFP:IL ratio = 6.5:3.5). Under the loading condition, the extracted response time was −0.8 s, and under the unloading condition, the response time was −0.5 s. The response rate of our sensor was relatively slow compared to previous IL-based pressure sensors or fabric-based pressure sensors (0.1–0.3 s) [42,43,44,45]. The relatively slow response characteristics were due to the viscoelastic property of the PVDF-HFP/IL film and the complicated weaving structure of the multifilament fiber electrodes on the fabric sheet [36,46,47,48]. However, considering the potential applications of PVDF-HFP/IL pressure sensors, such as monitoring of pressure distribution from a body, these response characteristics could be sufficient [36,48]. Additionally, the operation stability of the PVDF-HFP/IL pressure sensor was evaluated. Figure 5b shows the results from a cyclic test carried out for 1300 cycles of loading and unloading. As displayed, the sensor exhibited relatively stable operation up to 1300 cycles without noticeable degradation of the sensing performance. It should be noted that in the initial period of the cyclic test, the capacitance value in unloading states increased slightly and later stabilized. This can be attributed to the formation of a closer contact between the fiber electrodes and the PVDF-HFP/IL film due to repeated pressure.

### 3.5. Multi-Point Pressure Detection Using a 3 × 3 Pressure Sensor Array

**Figure 6:**
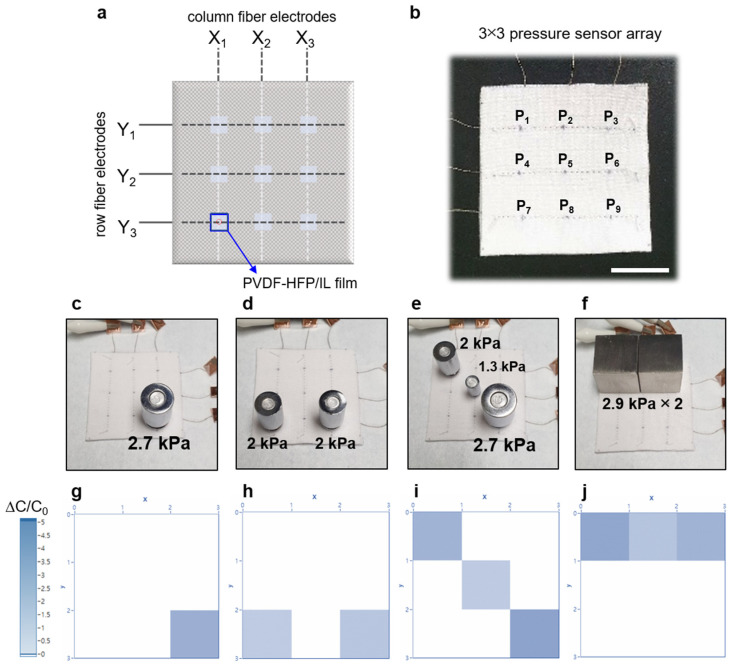
(**a**) The schematic layout, and (**b**) an optical image of a 3 × 3 PVDF-HFP/IL-based textile pressure sensor array. X_1_, X_2_, and X_3_ represent the column fiber electrodes, and Y_1_, Y_2_, and Y_3_ represent the row fiber electrodes (scale bar: 2 cm). (**c**–**f**) Optical images of the pressure sensor array loaded with various weights. (**g**–**j**) Corresponding pressure mapping data obtained from the sensor array.

To demonstrate the applicability of the PVDF-HFP/IL pressure sensors, a matrix-type 3 × 3 pressure sensor array was fabricated using polyester fabrics. Figure 6a,b show the schematic layout and optical image of the 3 × 3 pressure sensor array. Since the PVDF-HFP/IL-based pressure sensor had a cross-point structure, a matrix-type array could be simply constructed for multi-point detection. The column fiber electrodes were designated as X_n_ (*n* = 1,2,3), and the row fiber electrodes were designated as Y_n_ (*n* = 1, 2, 3) (Figure 6a). As shown in Figure 6b, each sensing point was designated as P_n_ (*n* = 1–9). Because the 3 × 3 pressure sensor array operated based on the capacitance variation, a multi-point pressure detection was possible. To demonstrate the multi-point pressure detection, one to three separate objects were placed at different sensing points on the sensor array (Figure 6c–f). The mapping of the capacitance values was carried out by using a homemade array tester composed of an LCR meter and a digital multimeter/switch system [36]. As shown in Figure 6g–j, the 3 × 3 sensor array could detect the positions and the weights of the objects without significant crosstalk between the sensing points. These results suggest that the textile pressure sensors implementing PVDF-HFP/IL film and multi-filament fiber electrodes could be successfully adopted in e-textile applications for multi-point detection.

## 4. Conclusions

In our study, we demonstrated a textile-based capacitive pressure sensor using a PVDF-HFP/IL composite film and multi-filament Ag-plated fiber electrodes. Using an optimized PVDF-HFP:IL ratio, we created a textile pressure sensor that exhibited a high pressure sensitivity, a wide detection range, and good operation stability. The PVDF-HFP:IL ratio had significant effects on the sensitivity and detection range of the sensor. The pressure-dependent capacitance variation was explained based on the change in the contact area, which was formed between the multi-filament fiber electrodes and the PVDF-HFP/IL film. To demonstrate the applicability and scalability of the PVDF-HFP/IL-based sensor, we used a 3 × 3 pressure sensor array; due to its matrix-type array structure and the capacitive-type sensing mechanism, identification of the different positions and weights of the objects was possible.
